# War against the Act

**Published:** 1913-11-22

**Authors:** 


### WAR AGAINST THE ACT.

### SCOTTISH DOCTORS RE-AFFIRM THEIR OPPOSITION.

A meeting of the Medical Guild (secretary, Dr. Fredk. Porter, 65 Morningside Road, Edinburgh), attended by over 100 doctors, was held in the Royal College of Physicians, Edinburgh, Dr. John Playfair, ex-President of the Royal College of Physicians, presiding.

Professor Russell moved: "That this Guild adheres to the position it adopted when the National Insurance Act came into operation, and it resolves to federate with all Associations which hold a similar policy—namely, the maintenance of the honour and freedom of the medical profession and the continuance of an uncompromising opposition to the method of administration of the medical benefits of the Act."

Professor Russell said the Guild consisted of members who for one reason or another were not able to join the medical panels in Edinburgh or elsewhere. Many doctors were compelled by financial considerations to become panel doctors, and many of these men felt towards the National Insurance Act as the members of the Guild felt. The freedom of the profession was the freedom of the public much more than the freedom of the profession. Under the Act the medical freedom of the public had been practically done away with.

The whole system of going compulsorily into the Act was abhorrent. It was a system of blackmail. If the Act had been truly philanthropic it would have dealt in the first place with the very poor, and the whole medical profession would have helped it. After Mr. Bonar Law's recent speech he felt satisfied that when there was a change of Government the new Government would seriously and earnestly, and as effectively as possible, deal with the Act in the direction of giving insured persons facilities in getting outside the Act, and freeing great institutions from the incubus of the Act.

Dr. James Carmichael, Edinburgh, in seconding the resolution, said the tendency of the Insurance Act was to degrade the medical profession.

Dr. Hart, Manchester, said they were now going to war, and he did not think there was any doubt of success. The reasons were loss of patients, the lowering of fees, and the gross iniquity of subjection to the Insurance Committee.

The resolution was adopted.

### Six Months' Panel Statistics.

An interesting light upon the working of medical benefit under the panel system has been shed by Dr. C. Fraser, of Brighton, who has published his statistics of his first six months' work. In the second quarter of this year he had 1,107 (average) on his panel list; in the third quarter he had 1,146 (average). These two quarters are, of course, much the healthiest time of the year; yet in April-June he paid 187 visits, and had 1,261 attendances at his surgery; and in July-September, 136 and 1,164 respectively. Even if the winter months proved to bring no greater work (though it is certain they must do), this experience is valuable as showing the amount of work a panel doctor undertakes in return for about £400 a year: namely, to see fifteen to sixteen patients a day, Sunday and week-day, including visits to the insured in their own homes. Consider how the work must be scamped by those who accept several thousand patients!

**Non-Panel Medical Societies.—The conference of non-panel medical associations announced in these columns last week will be held at the Waldorf Hotel, London, on November 29 at 3 p.m.**

